# Supplementary material for: Impaired Neutralizing Antibody Activity against B.1.617.2 (Delta) after Anti-SARS-CoV-2 Vaccination in Patients Receiving Anti-CD20 Therapy
Source: J Clin Med. 2022 Mar 21;11(6):1739. doi: 10.3390/jcm11061739 (PMC8952324; doi:10.3390/jcm11061739)

Supplemental Figure S1. Side effect questionnaire

1. Side effects after 1st vaccination:

◯ yes ◯ no

2. If yes, please mark the appropriate side effects, you had:

◯ local events (such as pain at injection site, redness, swelling)

◯ fever ◯ fatigue ◯ headache ◯ chills

◯ vomiting ◯ diarrhea ◯ muscle pain ◯ joint pain

◯ swollen lymph nodes ◯ others, such as ________________

3. In response to above mentioned side-effects, I took the following medication

◯ ____________________

1. Side effects after 2nd vaccination:

◯ yes ◯ no

2. If yes, please mark the appropriate side effects, you had:

◯ local events (such as pain at injection site, redness, swelling)

◯ fever ◯ fatigue ◯ headache ◯ chills

◯ vomiting ◯ diarrhea ◯ muscle pain ◯ joint pain

◯ swollen lymph nodes ◯ others, such as ________________

3. In response to above mentioned side-effects, I took the following medication

◯ ____________________

Supplemental Figure S2. Cutoff values for defining positivity for antibodies against different SARS-CoV-2 and common cold coronaviruses target epitopes by a bead-based multiplex assay.

**Target**	**Cutoff (MFI)**
SARS-CoV-2 Spike	6800
SARS-CoV-2 Spike S1	2700
SARS-CoV-2 Spike RBD	3800
SARS-CoV-2 Spike S2	3200
SARS-CoV-2 Nucleocapsid Protein	5900
HCoV-229E Spike S1	8012
HCoV-HKU1 Spike S1	4235
HCoV-NL63 Spike S1	4407
HCoV-OC43 Spike S1	3599
SARS-CoV Spike S1	41

MFI, mean fluorescence intensity; RBD, receptor-binding domain

Supplemental Figure S3. Radial Graphs of adverse reaction after first and second anti-SARS-CoV-2 vaccination of Rituximab treated patients and healthy controls.

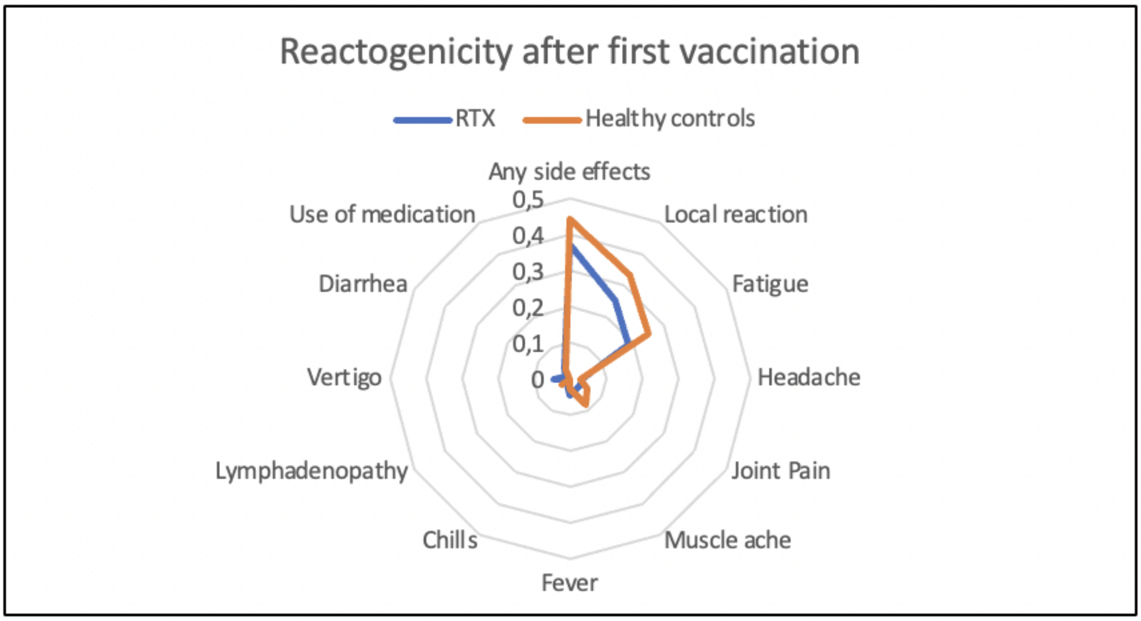

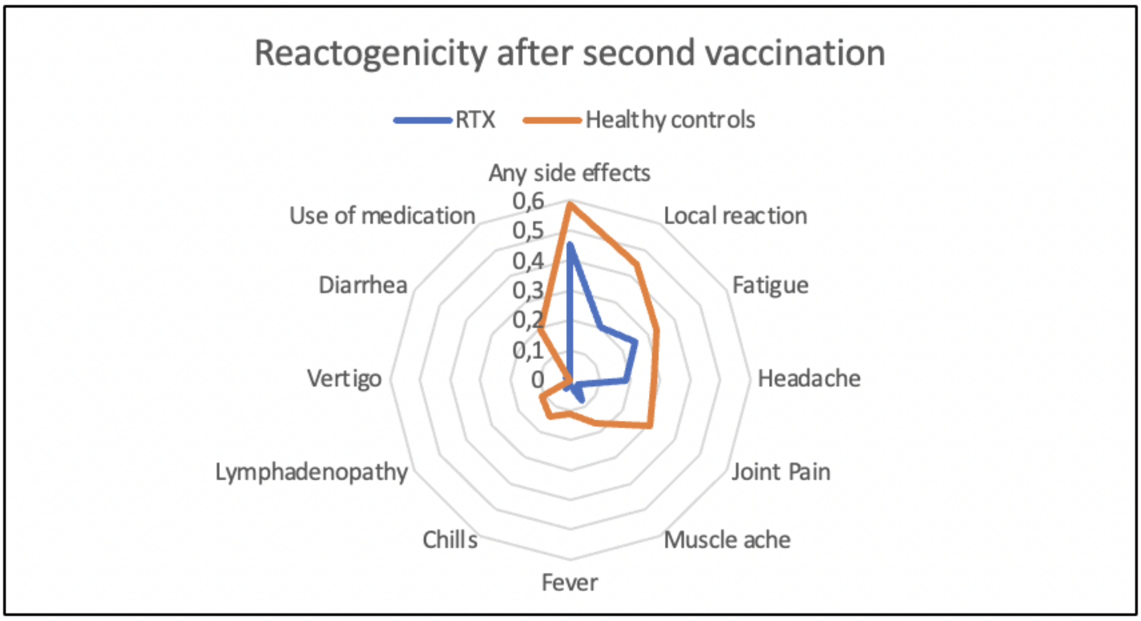

Supplemental Figure S4. Subgrouping of patients according to seroconversion of anti-S1 IgG, neutralizing antibodies, and anti-RBD antibodies.

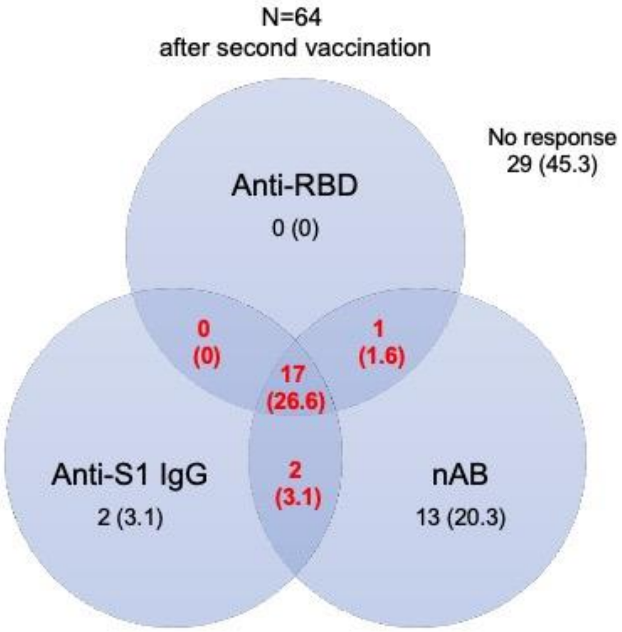

Supplemental Figure S5. CD4+ and CD19+ cell proportion of CD3+ lymphocytes, and correlation of CD4+ and CD19+ cells with anti-S1 IgG, neutralizing antibodies, and anti-RBD antibodies and neutralizing antibody activity against B.1.617.2 (delta).

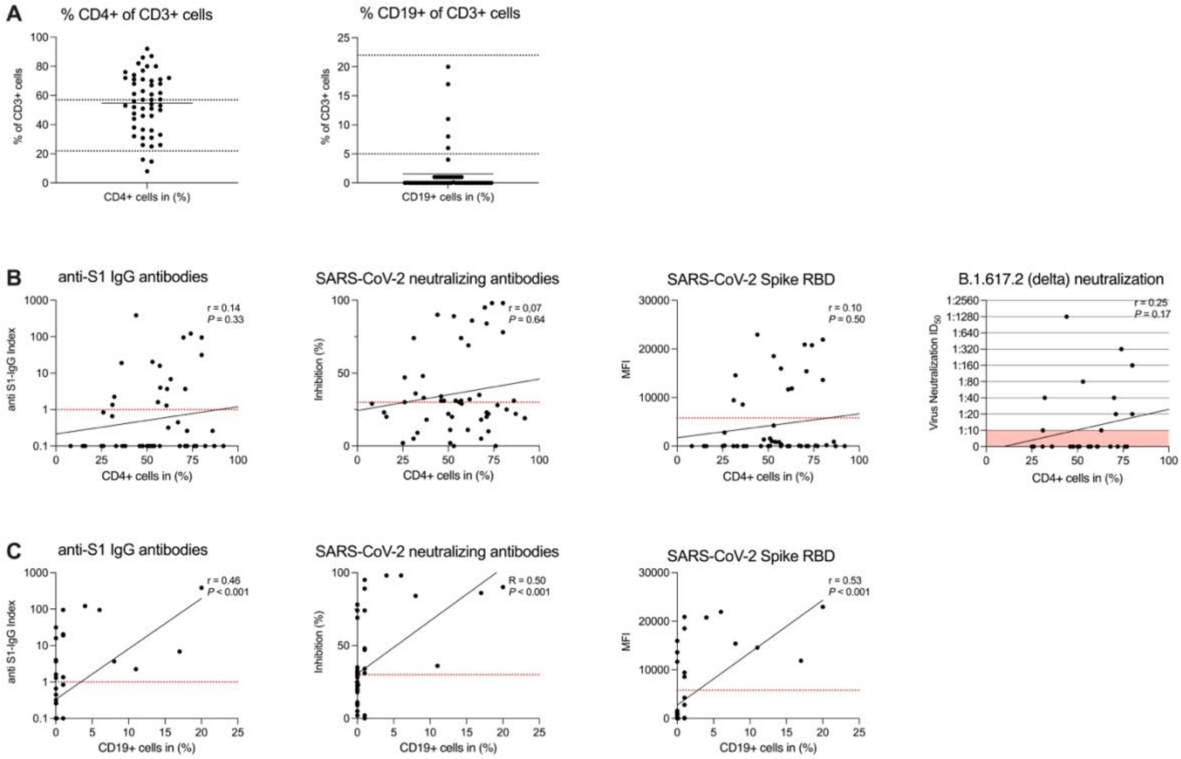

Supplemental Figure S6. Disease-specific humoral response and B-cell recovery depending on the last RTX treatment prior to the first anti-SARS-CoV-2 vaccination.

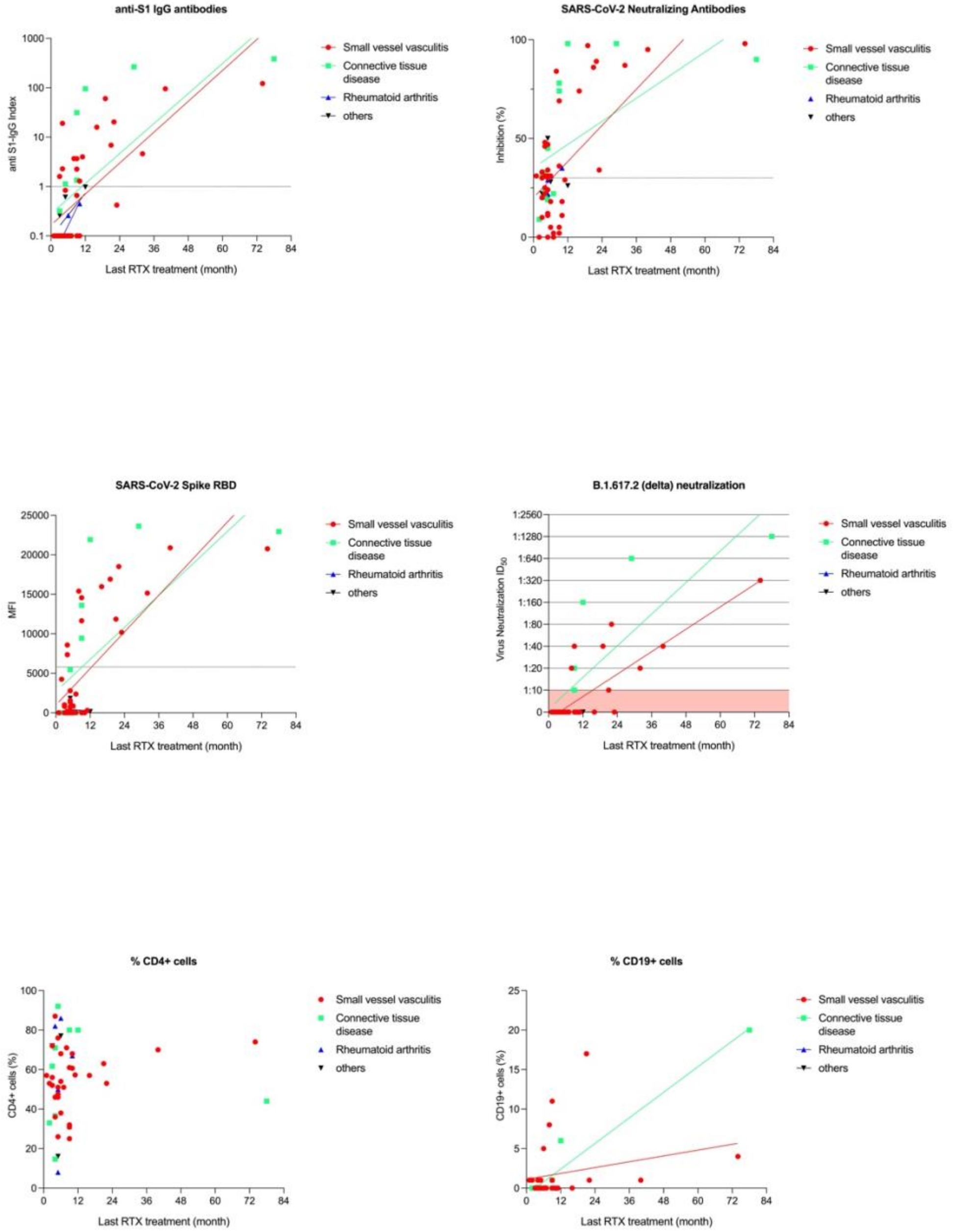

Supplemental Figure S7. Tretamnet-specific humoral response and B-cell recovery depending on the last RTX treatment prior to the first anti-SARS-CoV-2 vaccination.

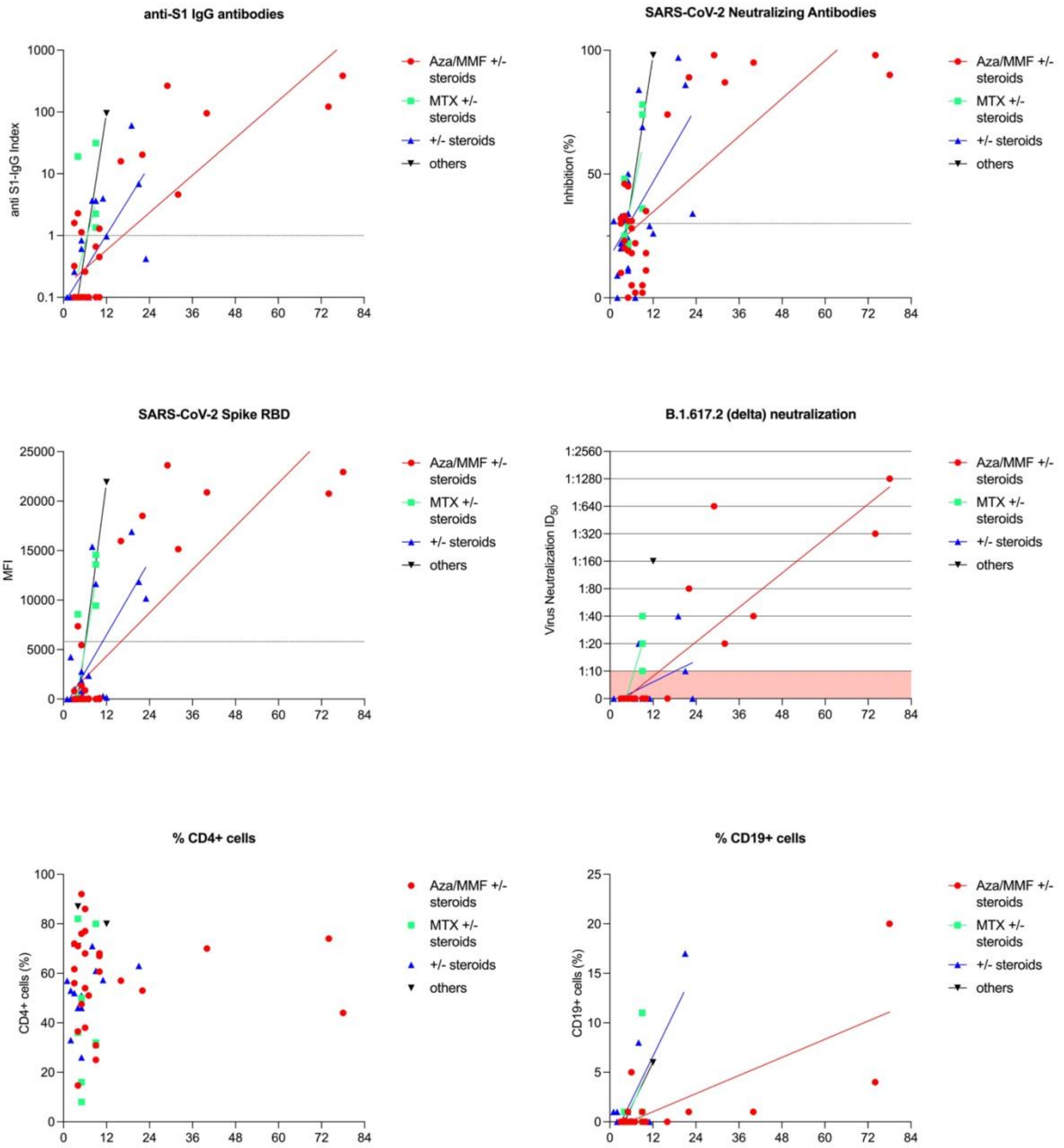

Supplement: Supplementary file 1 [file jcm-11-01739-s001.zip › jcm-1610549-supplementary.pdf]
